# P20 Clinical metagenomics from cell-free DNA: overview and lessons learned from 4 years as a clinical metagenomics provider

**DOI:** 10.1093/jacamr/dlad143.024

**Published:** 2024-01-03

**Authors:** Matthias Klaften, Ulrike Neder, Johannes Becher, Rafael Holzem, Sebastian Clanzett, Sonia Mazzitelli, René Hennig, Irene Gil-Farina, Sandra Unsleber, Silke Grumaz

**Affiliations:** Noscendo GmbH, Duisburg, Germany; Noscendo GmbH, Duisburg, Germany; Noscendo GmbH, Duisburg, Germany; Noscendo GmbH, Duisburg, Germany; Noscendo GmbH, Duisburg, Germany; Noscendo GmbH, Duisburg, Germany; Noscendo GmbH, Duisburg, Germany; Noscendo GmbH, Duisburg, Germany; Noscendo GmbH, Duisburg, Germany; Noscendo GmbH, Duisburg, Germany

## Abstract

**Background:**

Clinical metagenomics emerges more and more from academic research to clinical practice. Here, we share an overview of the field of clinical metagenomics on cell-free DNA found in the blood of patients with severe infections and share a few learning points regarding the implementation as a routine lab service and CE-IVD marked software running in the cloud.

**Methods:**

We use next-generation sequencing of cell-free DNA to digitally determine relevant pathogens from patients with systemic infections in blood and other bodily fluids using our CE-IVD marked DISQVER diagnostic platform. Until now we have collected microbial data from several thousand samples within 4 years of routine service.

**Results:**

The set up of the DISQVER test is within the regulatory framework of CE-IVD marking of software as a service (SaaS) and ISO 13485 accreditation. This brings practical implications on database quality, software lock-in/development/testing, change and risk management, technical documentation, requiring substantial resources. Also the wetlab process runs within tightly defined and controlled QM boundaries, pre-set by extensive analytical validations. We clinically validated our test for pathogen identification from blood within 8 clinical trials for intensive care medicine, haematology and coinfection in COVID-19, showing 2- to 3-fold higher identification rates for pathogens in comparison to standard of care blood culture. Furthermore, several case reports/ case series from different clinical backgrounds, frequently with difficult to diagnose pathogens, have been published. From the analysis of our routine processes with several thousand samples, we could show that the turn-around time of our mNGS process is very fast and can detect more species than blood culture. In particular, the relatively high proportion of fungi and dsDNA viruses indicates superiority of the method compared with the standard diagnostic pathway.

**Conclusions:**

To bridge the gap between academic research and clinical implementation, many aspects of regulatory, technical and data protection requirements need to be addressed. Within the timeframe of commercial availability DISQVER from cell-free DNA found in the blood of infected patients is increasingly becoming a useful tool for infection diagnostics.

